# Genetic and phenotypic characteristics of three Mainland Chinese families with choroideremia

**Published:** 2012-02-03

**Authors:** Qi Zhou, Liang Liu, Fei Xu, Hui Li, Yuri Sergeev, Fangtian Dong, Ruxin Jiang, Ian MacDonald, Ruifang Sui

**Affiliations:** 1 Department of Ophthalmology, Peking Union Medical College Hospital, Peking Union Medical College and Chinese Academy of Medical Sciences, Beijing, China; 2 National Eye Institute, National Institutes of Health, Baltimore, MD; 3 Department of Ophthalmology, University of Alberta, Alberta, Canada

## Abstract

**Purpose:**

To describe the phenotype and genotype of three Mainland Chinese families affected by choroideremia (CHM).

**Methods:**

Complete ophthalmic examinations were conducted in three unrelated Chinese families with CHM. Peripheral blood samples were collected from the families for genetic and immunoblot analysis. All exons and flanking intronic regions of the gene encoding Rab escort protein-1 (*Rep-1*) were amplified with PCR and screened for mutations with Sanger sequencing. The three-dimensional structure of mutated Rep-1 was modeled using sequence homology with rat proteins to analyze the effect of the mutation detected in one family.

**Results:**

All affected males had characteristic signs and symptoms of CHM; however, central visual acuity impairment occurred earlier than expected. All female carriers older than 45 years had pigmentary changes, and one female carrier was symptomatic with vision loss. Three different mutations in *Rep-1*, c.1801–1G>A, c.1130 T>A, and c.612delAG, were detected in the three families.

**Conclusions:**

In Mainland Chinese families, the central visual acuity of male patients with CHM can be affected at an early age (second decade), whereas female CHM carriers may manifest signs and symptoms at a later age (≥45 years). One previously reported and two novel *Rep-1* mutations were detected in three Chinese patients with CHM.

## Introduction

Choroideremia (CHM) is an X-linked progressive chorioretinal dystrophy [1]. Affected males experience night blindness in their teenage years, followed by visual field loss that leads to legal blindness later in life. Female carriers generally do not show serious visual impairment, but they may have patchy areas of pigmentation and retinal pigment epithelium (RPE) degeneration due to random X-inactivation, called lionization [2]. In most cases, CHM can be clinically diagnosed with fundus examination; however, because of interfamilial and intrafamilial variability in the clinical manifestations of CHM, modern molecular genetic testing is required to confirm the diagnosis [3].

The CHM gene has been mapped to Xq21.2 [4]. It consists of 15 exons spanning about 150 kb of genomic DNA and is expressed in many tissue types, including retinal photoreceptors, choroid, RPE, and lymphocytes [5]. CHM mRNA is about 5.6 kb long and encodes an intracellular protein of 653 amino acids, called Rab escort protein-1 (Rep-1), which is involved in the prenylation of Rab GTPases and intracellular vesicular trafficking [5].

At present, according to the Human Gene Mutation Database (HGMD), there are 113 reported mutations in the *Rep-1* gene, including missense/nonsense, splicing, small deletions, small insertions, small indels, gross deletions, and gross insertions. Reports of Asian CHM families with mutations are rare: Yip et al. [6] reported five mutations in five separate Chinese families; Fujiki et al. [7] reported 15 mutations in 18 Japanese families.

In this study, we describe the results of clinical and molecular analyses of three Mainland Chinese families with CHM. Two novel mutations as well as a previously reported mutation in *Rep-1* were identified.

## Methods

This study was approved by the Institutional Review Board of Peking Union Medical College Hospital and conformed to the tenets of the Declaration of Helsinki and the Guidance of Sample Collection of Human Genetic Diseases by the Ministry of Public Health of China. Informed consent was obtained from all subjects before entry into this study.

### Participants

Patients and their family members were seen in the Department of Ophthalmology, Peking Union Medical College Hospital. Except ocular diseases, they were generally healthy. Nothing remarkable was found in the medical history in all the participants. Complete ophthalmic examination was supplemented with optical coherence tomography (OCT; Topcon, Tokyo, Japan), visual field testing (Octopus 101), and full field electroretinography (ERG; RetiPort ERG system, Roland Consult, Wiesbaden, Germany). The ERG was recorded using corneal “ERG jet” contact lens electrodes under conditions that conformed to the standards of the International Society for Clinical Electrophysiology of Vision (ISCEV).

### Mutation screening of candidate genes

Peripheral bloods were drawn and collected in EDTA tubes. Samples were preserved in refrigerator before use. Genomic DNA was isolated from the leukocytes using a QIAmp DNA Blood Midi Kit (Qiagen, Hilden, Germany) according to the manufacturer’s protocol and kept in freezer. Genomic DNA was amplified from all exons and flanking intronic regions of the *Rep-1* gene with PCR [8]. Pairs of oligonucleotide primers specific for each exon were used to amplify the entire coding region of the *Rep-1*gene [3]. PCR products were directly sequenced on an ABI Prism 3730 Genetic Analyzer in both directions (Applied Biosystems, Foster City, CA).

### RNA analysis

After mutations were initially identified at the DNA level, a second fresh venous blood sample was obtained from members of family 1. Total RNA was extracted using a commercial kit (PAXgene Kit; Qiagen) according to the manufacturer’s instructions. Reverse Transcriptase PCR (RT–PCR) primers were chosen from exon 12 to exon 15. RT–PCR products were directly sequenced on an ABI Prism 3730 Genetic Analyzer.

### Immunoblot analysis

Proteins were extracted from a lysate of leukocytes from members of family 1. Proteins were separated with SDS–PAGE on 4%–20% gradient mini-gels and transferred onto a 0.45-µm nitrocellulose membrane (Invitrogen, Grand Island, NY) and then probed with mouse anti-human anti-Rep-1 antibody, clone 2F1 (Santa Cruz Biotechnology, City Santa Cruz, CA). Immunoreactivity was detected with a corresponding secondary anti-IgG antibody conjugated with horseradish peroxidase (Zymed Lab, Inc., San Francisco, CA) and imaged on X-ray film using SuperSignal West Pico or SuperSignal West Dura Chemiluminescent Substrates (Pierce, Rockford, IL).

### Molecular modeling

Molecular modeling of the Rep-1 structure and splice mutations was performed as described earlier [9]. Briefly, a model of human Rep-1 was built by homology modeling based on 2.2 crystal coordinates for the rat Rep-1 protein in a complex with monoprenylated Rab7 protein (PDB file: 1vg0) as the structural template [10]. Primary sequences were aligned with Needleman and Wunsch’s method [11], incorporated in the program Look, version 3.5.2 [12,13] for three-dimensional structure prediction. Finally, full-length monomeric Rep-1 and changes in protein structure corresponding to the detected gene mutations were built with the automatic segment matching method in the Look program [14] followed by 500 cycles of energy minimization. In the full-length Rep-1 structure, the elements of the structure corresponding to the unresolved fragments of Rep-1 were generated in a conformation that has not been justified experimentally, to show the possible location and structural role of these fragments in Rep-1. The same program generated the conformation of the protein with the detected mutation and refined this structure with 500 cycles of self-consistent ensemble optimization [13], which applies statistical mechanical mean-force approximation iteratively to achieve the global energy minimum structure. The geometry of the predicted structures was tested using the program Procheck [15].

## Results

### Clinical findings

**Figure 1:**
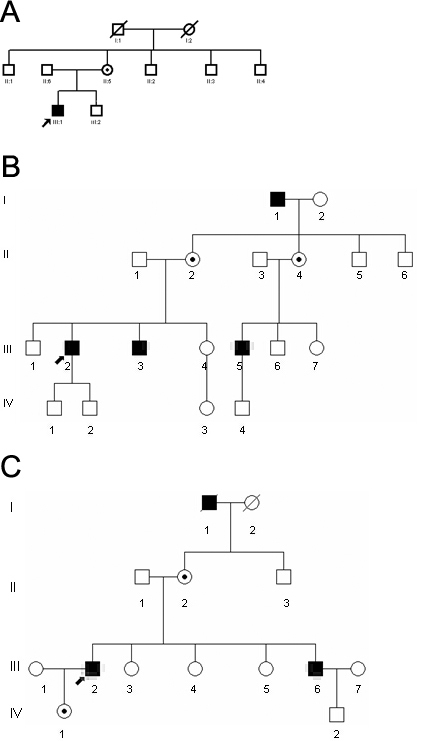
Family pedigrees of the three mainland Chinese choroideremia families. X-linked inheritance pattern is clear. Closed symbols indicate individuals with choroideremia (CHM) and open symbols indicate unaffected subjects. Dotted circles indicate female carriers. Arrows indicate probands. Slash indicates deceased person.

All pedigrees of the families showed X-linked transmission (Figure 1). Patient F1-III-1 and patient F3-III-2 were myopic. All affected male patients experienced poor night vision at an early age and decreased visual acuity in the third, fourth, and fifth decades. The patients’ clinical findings are listed in Table 1. One female carrier, F3-II-2, showed signs of decreased vision and had a reduced visual field, reduced ERG amplitudes, and fundus changes in the sixth decade. Carriers F1-II-2, F2-II-2, and F2-II-3 did not have any symptoms, but fundus changes were observed and documented with retinal photography (Table 2).

**Table 1:** Clinical features of patients with CHM.

**Patient**	**Age of decreased vision**	**Vision**	**Refraction**	**Visual field**	**Fundus**	**ERG**
F1-III-1	20s	OD:20/60 OS:20/100	−8 −9	tunnel vision	hypopigmented fundus choriocapillaris and RPE atrophy macula preservation	complete absence of rod responses and markedly reduced cone responses
F2-III-2	40s	OD:20/2000 OS:20/250	−9.25–1.50×50 −11.75–0.25×80			Extinguished ERG
F2-III-5	35s	OD:20/200 OS:FC	−1 plano			Extinguished ERG
F3-III-2	20s	OD:20/200 OS:20/50	−1.50–1.75×6 −0.25–1.75×1105	Central 1–2°	See Figure 6	Only low responses could be detected at 30HZ

**Table 2:** Clinical features of carriers with CHM.

**Patient**	**Age of test**	**Symptoms and signs**
F1-II-2	45	hypopigment changes in the midperiphery area fine RPE mottling patchy areas of chorioretinal atrophy (Figure 7A)
F2-II-2	71	NA; Fundus: diffuse punctate hypopigmentary changes (Figure 7B)
F2-II-3	67	NA; Fundus: diffuse punctate hypopigmentary changes
F3-II-2	69	Decreased vision Refraction:OD 0–3.50×130 20/60; OS +0.50+1.25×40 20/30; Fundus: see Figure 7C ERG: reduced rod and cone amplitude VF: constricted, central 20° VF
F3-IV-1	17	Refraction; OD −1.25–0.75×160 20/20; −1.00–1.00×8 20/20

### DNA analysis

**Figure 2:**
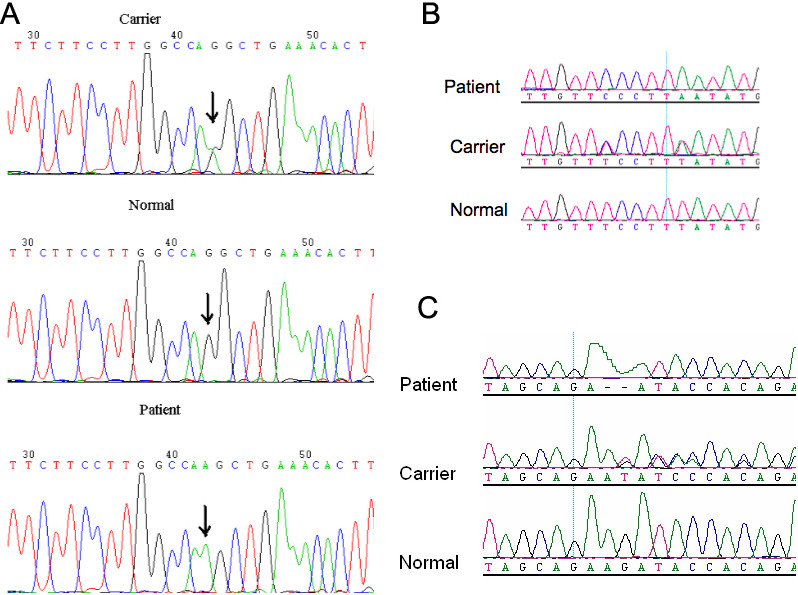
Electropherograms of the three families. The sequencing results show three different mutations. **A**: A c.1801–1G>A splice mutation was detected in family 1. **B**: A c.1130 T>A (Leu377Ter) mutation and a synonymous variation: c.1125 T>C were found in family 2. **C**: A c.612 del AG (p.Glu204GlufsX18) mutation was detected in family 3. All the carriers were heterozygous.

A transition mutation (G to A) at nucleotide position 1801–1 was detected in family 1, which is in the splice acceptor site of intron 14. A c.1130 T>A (Leu377Ter) mutation was detected in family 2, and a c.612 del AG (p.Glu204Glufsx18) mutation was detected in family 3. Carriers in the three families were heterozygous for these mutations. A synonymous variation, c.1125 T/C (Phe375Phe), was also found in family 2 (Figure 2).

### Real-time polymerase chain reaction analysis

**Figure 3:**
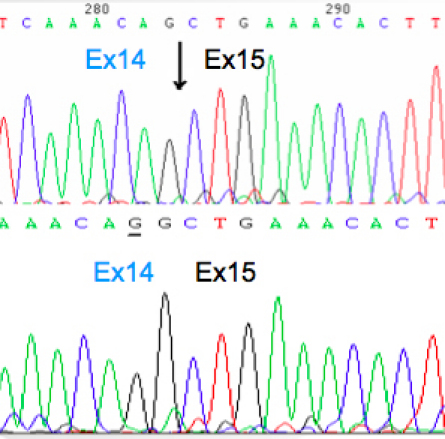
Reverse-transcriptase (RT)-PCR analysis. Sequencing results from the proband of family 1 show a deletion of G on the mutated exon 14 (upper sequence). Arrow indicates the border between exon 14 and exon 15.

Sequencing of the RT–PCR product from family 1 revealed that G is missing from cDNA position 354 of a family 1 patient. This is as a result of the altered splice site at this location (Figure 3).

### Western blot

**Figure 4:**
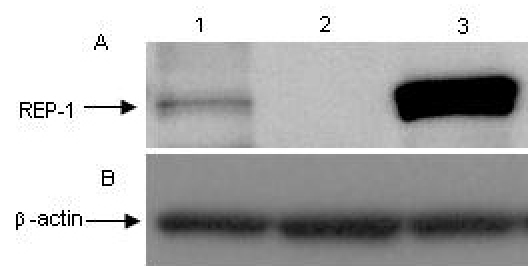
Western blot results from the family 1 show the absence of Rep-1 protein in the patient (line 2) and a reduced amount of Rep-1 in the female carrier (Line 1). Lane 3 is the normal brother of the patient. **A**: Mouse anti-REP-1, clone 2F1 demonstrated the absence of Rep-1 protein in the patient and a reduced amount of Rep-1 protein in the female carrier. **B**: β-actin antibody was used as a loading control to ensure an adequate protein sample in each lane.

Western blot analysis was performed on protein samples from members of family 1, the patient, his mother, and unaffected brother. Blot incubation with mouse anti-Rep-1, clone 2F1, demonstrated the absence of any Rep-1 protein band in the patient and showed a reduced amount of the Rep-1 protein in the female carrier when compared to the band intensity of the unaffected brother (Figure 4).

### Molecular modeling

**Figure 5:**
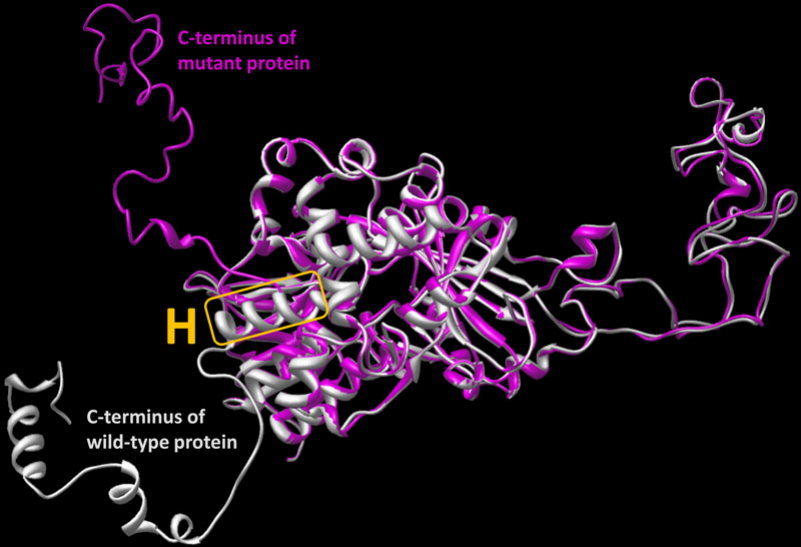
The effect of the downstream cryptic splice site on a three-dimensional structure of the Rep-1 protein is shown. The mutation cause changes in the last 63 amino acid residues at the C-terminus. The superposition of ribbon structures for wild-type and mutant proteins are shown in light gray and magenta, respectively. In the wild-type protein, the α-helix that might be critical for maintaining global protein stability is included in the orange box and labeled with H.

The downstream cryptic splice site detected in family 1 causes changes in the last 63 amino acid residues at the C-terminus of the protein. This change will affect the conformation of Rep-1 at the C-terminus by decreasing the content of the regular α-helical structure as shown in Figure 5. Based on molecular modeling, the changes caused by the splice mutation might destabilize the structure of the helix (orange H) that potentially could affect the global stability of the Rep-1 protein (Figure 5).

## Discussion

**Figure 6:**
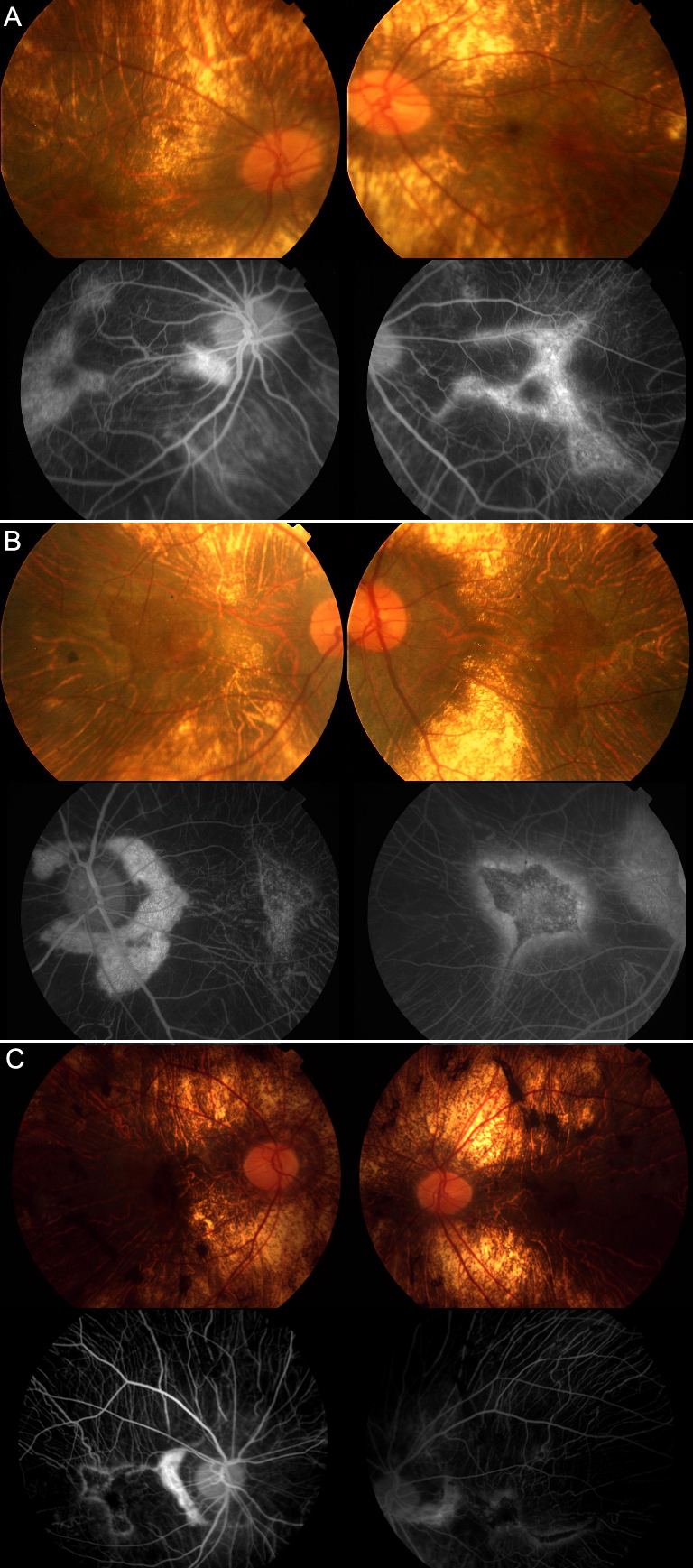
Fundus photographs and fluorescein fundus angiography from choroideremia (CHM) patients. Characteristic retinal and choroidal atrophy and relatively preserved macular area are revealed. (**A**) F1-III-1, (**B**) F2-III-2, and (**C**) F3-III-2.

This report is the first to describe genotype/phenotype findings in Mainland Chinese families affected with choroideremia. Clinical findings of the male patients included early-onset night blindness, constricted visual fields, reduced ERG rod and cone response amplitudes, as well as chorioretinal atrophy consistent with the diagnosis of CHM (Figure 6). For the males affected with CHM in our study, central vision loss occurred in the third, fourth, and fifth decades, which is much younger than reported central vision loss occurring between the sixth and eighth decades [16]. Roberts et al.’s study of 115 CHM males (mean age: 39 years) showed that 84% of males under the age of 60 years had visual acuities of 20/40 or better and 35% of those over 60 years of age had visual acuities of 20/200 or worse, suggesting a slow rate of visual acuity loss and a generally good prognosis for retaining central visual acuity until the seventh decade [17]. An Internet-based survey of patients with CHM with responses mainly from North America, presumably of largely Caucasian origin (unpublished data from Qi Zhou and Ian MacDonald), found that 60% of respondents had non-functional vision (defined as vision less than 20/400 OU and field less than 5 degrees) and were between 50 and 70 years of age. This result raised questions regarding whether our observations are related to ethnicity or other modifiers affect the rate of vision loss. Our study does not answer these questions definitively; further studies on the CHM phenotype in Chinese families are needed. We should, however, consider the possibility that Chinese patients with CHM may experience severe vision loss at an earlier age than those of Caucasian origin.

**Figure 7:**
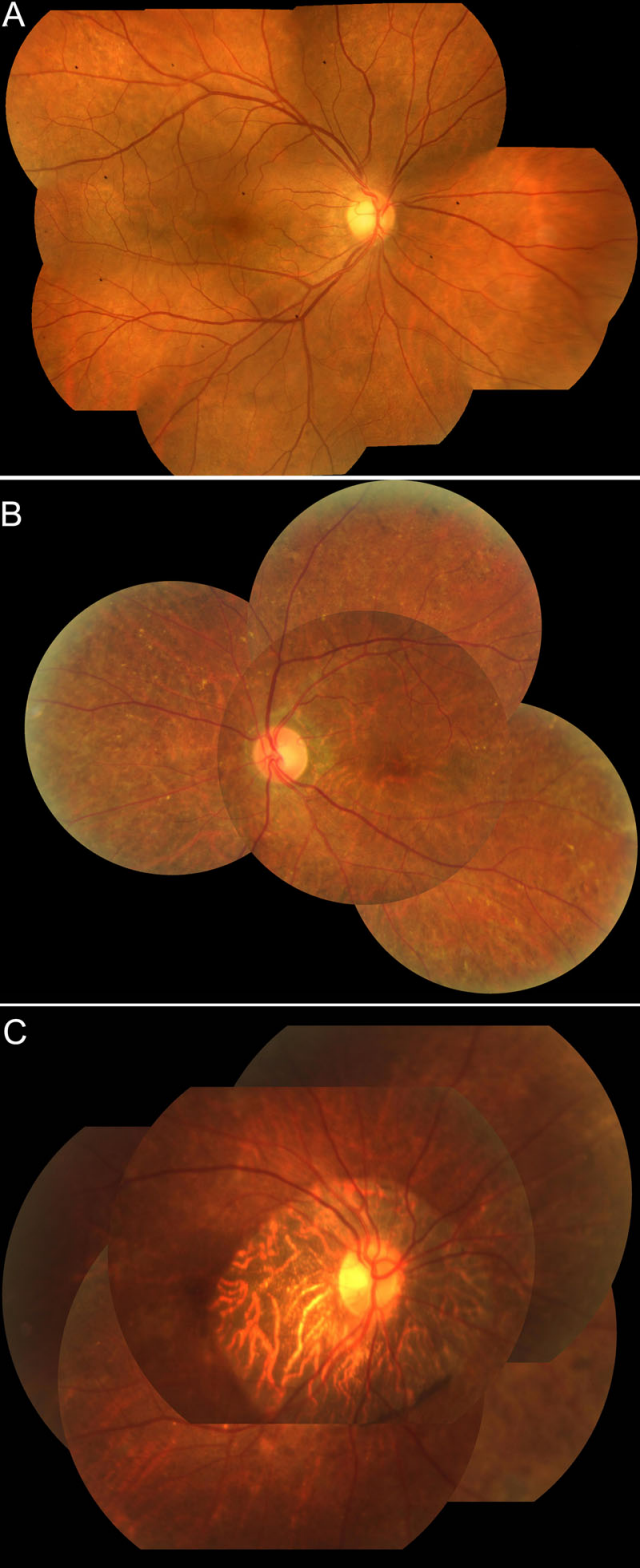
Fundus photographs of female carriers. **A**: (F1-II-2) Fine pigment mottling in the midperiphary retina. **B**: (F2-II-2) Pigmentary clumping changes are found in the posterior pole and peripheral retina. **C**: (F3-II-2) A ring of retinal and choroidal atrophy is found around the optic disc.

In our study, the female carriers in families 1 and 2 were asymptomatic but had pigmentary changes in the fundus. One older female carrier (age 69) in family 3 showed signs and symptoms similar to affected male patients (Figure 7). This may be due to skewed X-chromosome inactivation. Even though female carriers are generally asymptomatic, careful fundus examination can reveal signs of chorioretinal degeneration after the second decade [16]. Results (to be published) from the Internet-based survey (Qi Zhou and Ian MacDonald) indicate that age is a significant factor in predicting symptoms and signs in female CHM carriers. In family 3, the 69-year-old carrier showed significant CHM signs and symptoms, while the younger carrier (age 17) did not show any signs and symptoms. The difference in phenotype between the two carriers is likely related to age and the progressive nature of signs in female carriers. Compared to Caucasian patients, Chinese patients have significantly more pigment in the RPE and choroid, making detecting fundus changes in asymptomatic Chinese patients more difficult; therefore, asymptomatic but mildly affected females are likely under-reported. Since signs and symptoms manifest with time, careful examination and close follow-up should be conducted in CHM carriers.

Sequence analysis of the 15 exons and adjacent splice sites of the *Rep-1* gene allows for detecting mutations in approximately 60%–95% of affected males [16]. Using this strategy, three different mutations were detected in three Chinese families; carriers in each family were heterozygous for these mutations. A previously reported G to A transition mutation (c.1801–1) was found in the *Rep-1* gene of family 1; and RT–PCR revealed a G deletion in the resulting cDNA. This transition at c.1801–1 alters the invariant AG dinucleotide of the acceptor splice site in intron 14 leading to changes in the protein sequence involving the 63 carboxy terminal amino acid residues. In silico analysis showed that, as a consequence, the mutant protein lacks the entire C-terminal helix of the wild-type protein. The C-terminal fragment has a crucial role in Rep-1 function [19]. Recombinant rat Rep-1 protein lacking the 70 C-terminal amino acids does not have the ability to assist in the prenylation of Rab proteins [9,18]. The Rep-1 C-terminus functions as a mobile lid that covers a conserved hydrophobic patch on the surface of Rep-1 that binds the C-terminus of Rab [19]. The lack of a Rep-1 band in the immunoblot analysis of protein from the patient with CHM and the reduced Rep-1 band intensity in the carrier lane could result from the absence of the protein or changes in its affinity for the monoclonal anti-Rep-1 antibody. We predict this mutation in the splice site may cause Rep-1 to be unstable and easily digested by the cell’s proteolytic system or may give an effective complete loss of protein through the nonsense mediated mRNA decay.

Two novel mutations, c.1130 T>A (p.Leu377Ter) and c.612 del AG (p.Glu204Glufsx18), were detected in family 2 and family 3, respectively. The substitution c.1130 T>A is a nonsense mutation expected to truncate the protein. The deletion c.612 del AG is predicted to produce a frameshift and a premature termination codon, 18 codons downstream from the mutation. Both mutations result in truncation of the Rep-1 protein. A truncation at p.Leu377 would significantly disrupt the structure of Rep-1 Domain I that contacts Rab proteins, thereby rendering Rep-1 not only dysfunctional but also likely structurally unstable. The truncation at p.Glu204 would further result in the loss of prenyl binding capabilities and RabGGTase function due to the complete lack of Rep-1 Domain I in addition to Domain II disruption. Immunoblot analysis of the protein from these two families was not performed because of the nature of the mutations. A polymorphism, c.1125 T/C (p.Phe375Phe), was also found in family 2; however, it does not lead to any amino acid change and is therefore pathologically irrelevant.

Few important genotype-phenotype correlations have been demonstrated in patients with CHM. Individuals with full deletions of the *Rep-1* gene seem to be no more adversely affected than those with point mutations that will result in a truncated, unstable protein [16]. In the families reported here, all affected males had a similar range and severity of symptoms and experienced early central visual acuity loss. One female carrier in family 3, with a mutation c.612 del AG (p.Glu204Glufsx18) resulting in a shorter Rep-1 protein than in families 1 and 2, had more severe CHM symptoms and signs. Does a mutation resulting in a short peptide containing only the N-terminal 204 amino acids of Rep-1 lead to an early-onset disorder with more severe symptoms in female carriers? Further examples may answer this question. As age seems critical to the onset of signs and symptoms of CHM, the younger carrier in family 3 should be followed up closely. Due to the nature of her *Rep-1*mutation, she may develop early and severe symptoms and signs resembling those of her older relative.

The findings from our study of three Mainland Chinese CHM families add to the spectrum mutations in the *Rep-1* gene and will certainly aid in future mutation screening and genetic counseling in Chinese patients.
